# Insufficient S-adenosylhomocysteine hydrolase compromises the beneficial effect of diabetic BMSCs on diabetic cardiomyopathy

**DOI:** 10.1186/s13287-022-03099-1

**Published:** 2022-08-13

**Authors:** Ying Wang, Yuying Zhang, Kegong Chen, Jie Liu, Donghong Wu, Yao Cheng, Hongjie Wang, Yanbo Li

**Affiliations:** 1 Department of Endocrinology, First Affiliated Hospital of Harbin Medical University, Harbin, People’s Republic of China; 2 Department of Endocrinology, First Hospital of Harbin, Harbin, People’s Republic of China; 3 Department of Pathology, First Hospital of Harbin, Harbin, People’s Republic of China; 4 Department of Thoracic Surgery, First Affiliated Hospital of Anhui Medical University, Hefei, Anhui People’s Republic of China; 5 Future Medical Laboratory, Second Affiliated Hospital of Harbin Medical University, Harbin, People’s Republic of China; 6 Department of Endocrinology, Forth Affiliated Hospital of Harbin Medical University, Harbin, People’s Republic of China; 7 Department of Endocrinology, South China Hospital of Shenzhen University, No. 1 Fuxin Road, Longgang District, Shenzhen, 518116 People’s Republic of China

**Keywords:** Diabetic cardiomyopathy, BMSC, Stem cell, Oxidative stress, SAHH

## Abstract

**Background:**

Autologous stem cell therapy is a promising strategy for cardiovascular diseases including diabetic cardiomyopathy (DCM), but conclusions from clinical trials were compromised. We assumed that diabetes might induce the dysfunction of stem cells and thus limit its therapeutic effect. This study aimed to compare the effect of diabetes and nondiabetes-derived bone marrow mesenchymal stem cells (BMSCs) transplantation on DCM and explored the potential mechanism.

**Methods:**

Rats with diabetes were induced using high-fat diets and streptozotocin (STZ) injection. BMSCs harvested from diabetic and nondiabetic rats were infused into DCM rats, and the effects on the heart were identified by echocardiography and histopathology. The inhibition or overexpression of SAHH in nondiabetic and diabetic BMSCs was used to confirm its key role in stem cell activity and cardiac therapy.

**Results:**

Compared with normal BMSCs, the therapeutic effects of diabetic rat-derived stem cells on improving cardiac function and adverse remodeling were significantly attenuated. In vitro, diabetic BMSCs had lower cell viability and paracrine function than nondiabetic BMSCs. It was further found that diabetic BMSCs had obvious mitochondrial oxidative stress damage and S-adenosylhomocysteine (SAH) accumulation due to S-adenosylhomocysteine hydrolase (SAHH) deficiency. SAHH inhibition by adenosine dialdehyde (ADA) or shSAHH plasmid in normal BMSCs significantly reduced the favorable effects on endothelial cell proliferation and tube-forming capacity. In contrast, SAHH overexpression in diabetic BMSCs significantly improved cellular activity and paracrine function. Transplantation of BMSCs with SAHH overexpression improved cardiac adverse remodeling and angiogenesis. Activation of the Nrf2 signaling pathway may be one of the key mechanisms of SAHH-mediated improvement of stem cell viability and cardiac repair.

**Conclusions:**

Diabetes leads to compromised bioactivity and repair capacity of BMSCs. Our study suggests that SAHH activation may improve the cardioprotective effect of autologous transplantation of diabetes-derived BMSCs on patients with DCM.

**Graphical abstract:**

Diabetes induced the inhibition of S-adenosylhomocysteine (SAH) expression and aging phenotype in BMSCs and thus decreased the cell viability and paracrine function. Compared with normal BMSCs, the therapeutic effects of diabetic rat-derived BMSCs on improving cardiac function and adverse remodeling were significantly attenuated. SAHH overexpression in diabetic BMSCs significantly rescued cellular function partly via activating Nrf2/HO-1 signal. Transplantation of diabetic BMSCs with SAHH overexpression improved angiogenesis and cardiac adverse remodeling in rats.
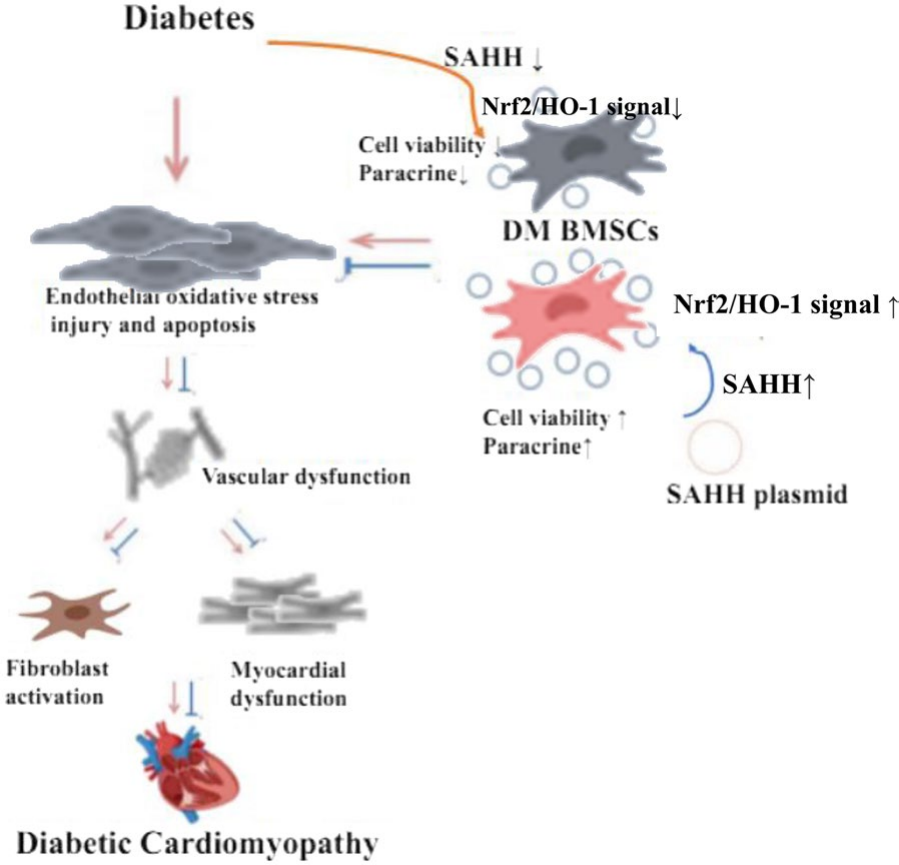

**Supplementary Information:**

The online version contains supplementary material available at 10.1186/s13287-022-03099-1.

## Background

The prevalence of diabetes mellitus has continued to increase over the past several decades [1]. Type 2 diabetes mellitus (T2DM), which accounts for 90–95% of diabetic cases, is characterized by insulin resistance and glycolipid metabolism disorder [2]. T2DM is a strong cause of the development of heart failure independent of coronary artery disease, hypertension, and valvulopathy, which was termed diabetic cardiomyopathy (DCM) [3]. Although there is an exponential increase in the number of preclinical and clinical studies on DCM over the past years, there is no validated therapy to improve diabetic cardiomyopathy [4].

Bone marrow mesenchymal stem cells (BMSCs) represent the most important source for regenerative medicine with an excellent capacity for multipotential differentiation and self-renewing [5]. Besides, the paracrine effects related to the secretion of various cytokines promote the repair of surrounding tissue cells. Accumulated preclinical studies have confirmed that BMSCs improve heart dysfunction and revascularization post-infarction [6–8]. Various types of stem cells, including BMSCs and adipose tissue-derived stem cells, had been demonstrated promising therapeutic candidates for the treatment of diabetic cardiomyopathy and other diabetic complications [5, 9–11]. Although the benefits remain controversial [12, 13], autologous BMSCs transplantation remained the most popular candidate for clinical translation to treat DCM due to the advantages of ethics, immunogenicity, and safety [14]. Investigating the potential strategy to improve the treatment benefits was important.

MSCs derived from diabetic patients or animals have high senescence characteristics, including the functional decline of cell proliferation, differentiation, and paracrine effects [15]. In recent years, studies have confirmed that diabetic stress induces a decreased activity of stem cells derived from the nerve, skeletal muscle, and adipose tissue, in which oxidative stress is one of the key mechanisms [9, 16]. BMSCs are the primary self-donor for the treatment of heart disease. Whether BMSCs derived from diabetes have a decreased activity in stem cell is still unclear. How improve the biological activity of BMSCs derived from diabetic patients is of great significance to promote the transformation and application of autologous stem cells in diabetic cardiomyopathy.

Oxidative stress is a common key factor affecting the progression of DCM and the aging of stem cells. In this study, we compared the cardioprotective effects of diabetic and normal rat-derived BMSCs on DCM and explored the underlying mechanisms. We found that BMSCs from nondiabetes but not from diabetes rats have a significant therapeutic effect on the diabetic heart. The disorder in S-adenosine homocysteine metabolism of BMSCs from DM is the key catalyst of oxidative stress, which is of great significance to clinical translation.

## Methods

### Experimental animals

The experimental rats model with type 2 diabetes mellitus was established as previously described [17]. Sprague Dawley male rats (8 weeks old) were randomly divided into normal control and DCM groups (purchased from the Animal Centre of Second Affiliated Hospital of Harbin Medical University). Normal rats feed on the normal chow diet containing 4 % kcal derived from fat. Rats in the DCM group were provided high-fat diets (HFD; 60% kcal from fat, Research Diets) for 8 weeks [18]. Then, HFD-fed rats were administrated with a single dose of 25 mg/kg streptozotocin (STZ, intraperitoneal injection, dissolved in 10 mmol/l citrate buffer, pH 4.5, Sigma-Aldrich, Saint Louis, MO) to induce type 2 diabetes. Blood glucose was consecutively measured by monitoring tail capillary blood glucose levels weekly. Rats with more than three random glucose level measurements ≥  6.7 mmol/l were considered diabetic status [11]. Then, 12 weeks after STZ injection, the DCM model was assessed and confirmed by echocardiography. The study protocols were approved by the Committee on Animal Care of Harbin Medical University.

About 2 × 10^6^ BMSCs were suspended in 300 µl sterile physiological saline and transplanted through tail-vein infusion in diabetic rats at 8 weeks after STZ injection [19]. The BMSC infusion was conducted every week, 4 times in all. The normal rats and DCM rats were infused with 0.3 mL sterile physiological saline as a control group. As mentioned in our recent study [20], recipient rats were intraperitoneally injected with cyclosporin A 5 mg/kg/day from 3 days before BMSCs treatment.

### The isolation, cultivation, identification, and transfection of BMSCs

As previously described, BMSCs were isolated from bone marrow stroma harvested from the femurs and tibias which were harvested from nondiabetic and diabetic rats (8 weeks after STZ injection), respectively [21]. BMSCs were cultivated in Dulbecco's modified Eagle's medium (DMEM)-F12 (HyClone) containing 10% fetal bovine serum (FBS, Gibco), 5% CO2 at 37 °C. BMSCs from the fifth passage were used for phenotypical identification, cardiac transplantation, and co-culture assays in vitro. BMSCs were treated with adenosine deaminase (ADA, 30 μmol/L) for 24 h or transfected using plasma with shSAHH sequence for 48 h to inhibit SAHH expression [22].

### HUVECs culture and treatment

Human umbilical vein endothelial cells (HUVECs) were cultured in DMEM medium (HyClone) supplemented with 10% fetal bovine serum, and 5% CO2 at 37 °C in humidified conditions [23]. HUVECs were treated with 200 μM palmitic acid (PA) and 25 mM glucose for 24 h to mimic the diabetic microenvironment [24].

### Flow cytometry analysis

Cultured BMSCs were digested using 0.25% trypsin and terminated with DMEM containing 10% FBS. After centrifugation at 1500 rpm for 5 min, the pellet was suspended and washed with PBS buffer. About 2 × 10^6^ BMSCs suspended with 100 μL of PBS were incubated with antibodies against surface markers of CD29, CD90, CD105, CD34, CD45, and CD11b for 30 min [17]. Cells were washed and analyzed via flow cytometry (BD Biosciences, USA). Unlabeled BMSCs were treated as a negative control.

### Echocardiography

As described in our previous study [20], echocardiography was used to assess heart function using an ultrasound system (Philips-EPIQ 5). The left ventricular end-diastolic diameter (LVIDd) and left ventricular end-systolic diameter (LVISd) were measured by parasternal short-axis scans at the papillary muscle level, and the left ventricular ejection fraction (LVEF) and left ventricular shortening fraction (LVFS) were calculated using computer algorithms simultaneously. Doppler was applied to assess the early-to-late diastolic mitral annular velocity (E′/A′) ratio.

### Histological analysis

The cardiac tissues were fixed in 4% paraformaldehyde (pH 7.4) for several days, embedded in paraffin, and sectioned (5 mm thick slices) for histological hematoxylin–eosin, Masson trichrome, and Sirius Red staining [25]. Histological images were obtained using a light microscope (Olympus, Japan) with 200× magnification.

### Measurement of MDA content

Cellular or cardiac malondialdehyde (MDA) contents were measured using a commercial kit (Cat#S0131, Beyotime) in line with the manufacturer’s protocol as described previously [25].

### Measurement of ROS content

ROS contents were evaluated by DHE staining (Cat#S0063, Beyotime) in vivo and in vitro following the manufacturer’s instructions [26]. Fresh heart slices or cultured cells were stained with DHE solution (5 μmol/L) at 37 °C for 30 min away from the light and then visualized under an Olympus fluorescence microscope.

### Cell viability

Cellular viability was determined using the commercial CCK-8 assay kit (Cat#C0038, Beyotime) according to the manufacturer’s instructions [26].

### ELISA analysis

VEGF, bFGF, SAH, and Hcy levels in the medium of BMSCs were determined using commercial ELISA kits (kselisa and cusabio, China) according to the manufacturer's instructions [20].

### JC-1 staining

Mitochondrial membrane potential was measured using a commercial JC-1 assay kit (Beyotime, China) [27].

### Edu staining

HUVECs were co-cultured with BMSCs for 24 h under a Transwell chamber system. The HUVEC proliferation was assessed using a commercial EdU Kit (UE, China) in line with the manufacturer’s protocols as previously described [20].

### Assessment of tube formation

The tube formation assay of HUVECs was evaluated using Matrigel Basement Membrane Matrix (100 µl/well; Corning, Standard, USA) as previously mentioned [20]. The average number of tubules and dendritic length was calculated using ImageJ software.

### Immunofluorescence

As described in our previous study [20], heart slices were blocked by goat serum for 1 h and then incubated with antibodies α-smooth muscle actin (α-SMA, AF1032, Affinity) and vWF (ab6994, Abcam) to assess angiogenesis. After staining with secondary antibodies, the images were obtained using a fluorescence microscope (Leica, Germany).

### Western blot

Total protein was obtained using RIPA lysis buffer (Beyotime, China) from BMSCs after treatment if necessary. Protein contents were estimated using a BCA Protein Assay Kit (Beyotime, China). Protein samples were separated by 10% SDS-PAGE and transferred to 0.22 μm PVDF membrane. The membrane was incubated with primary antibodies overnight at 4 °C, with anti-SAHH (10757, Proteintech, Wuhan, China), NRF2 (66504, Proteintech, Wuhan, China), KEAP1 (60027, Proteintech, Wuhan, China), HMOX1 (66743, Proteintech, Wuhan, China), SOD1 (67480, Proteintech, Wuhan, China), SOD2 (66474, Proteintech, Wuhan, China), and p67phox (15551, Proteintech, Wuhan, China). The bands were incubated with HRP-conjugated secondary antibodies for 1 h and visualized by a chemiluminescence imaging system (Tanon, China) [28].

### qRT-PCR

Total RNA from heart tissue was extracted using TRIzol Reagent according to the manufacturer's protocol (ThermoFisher, USA), and the harvested RNA was reverse-transcribed into cDNA. Further, cDNA was amplified using the real-time PCR method (Sequence Detection system, Bio-Rad, USA). The primer sequences of *Nppa, Nppb, and Myh7* used are listed in Additional file 4: Table S1, and the commercial β-actin primer was obtained from Sango Biotechnology (Shanghai, China). The transcript level of each gene was normalized by β-actin.

### Statistical analysis

All data are presented as means ± standard deviation (SD). The statistical analysis and visualization were conducted by GraphPad Prism (version 6.0). The data were analyzed by one-way analysis of variance or Student's t test. A *P* value less than 0.05 was considered statistically significant.

## Results

### The distinctive effect on diabetic cardiomyopathy of BMSCs harvested from health and diabetic donors in vivo

**Fig. 1:**
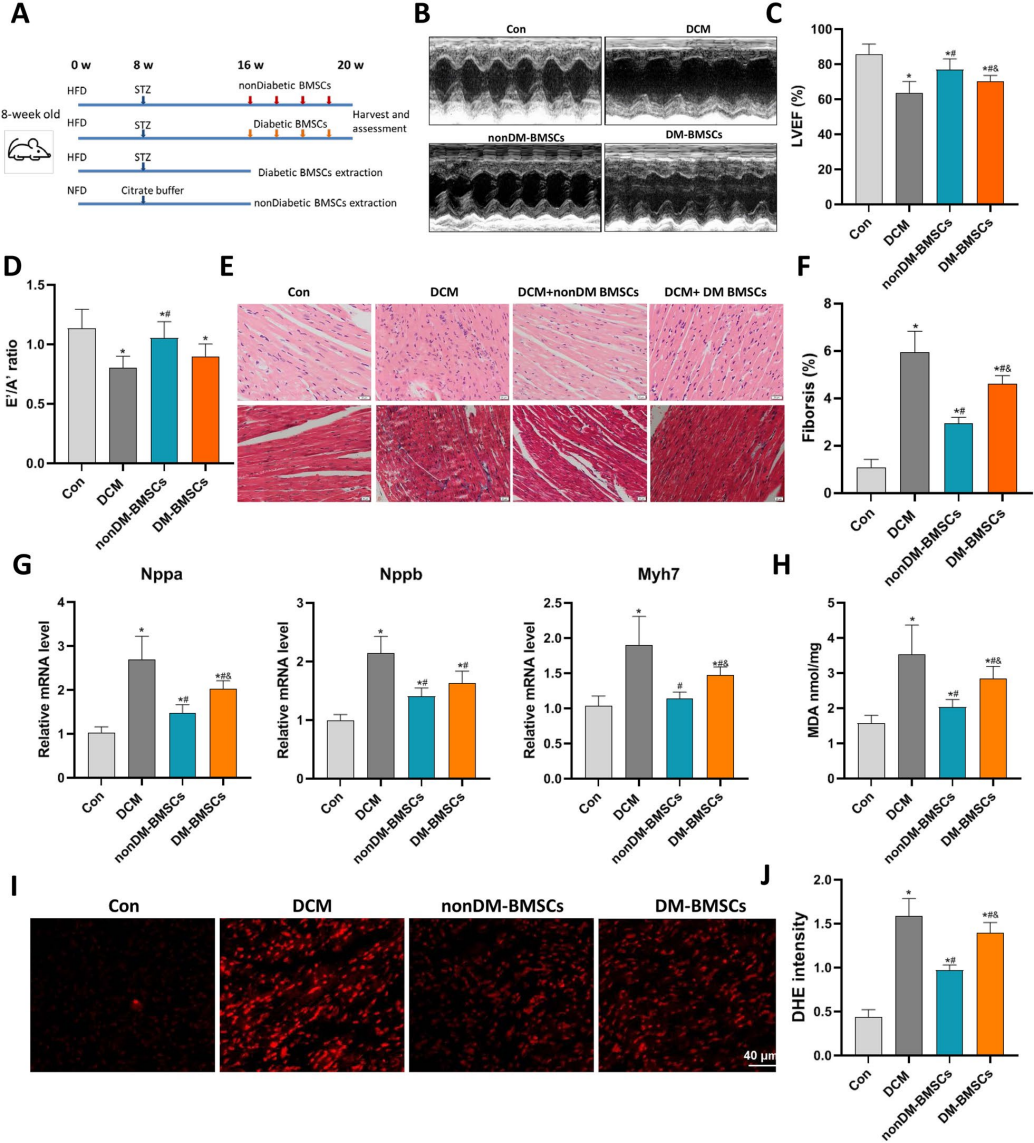
The distinctive effect on diabetic cardiomyopathy of BMSCs harvested from healthy and diabetic rats. BMSCs were infused 4 times, and cardiac function was measured using echocardiography (**a**) in control and DCM rats. The LVEF (**b**) and E′/A′ ratio (**c**) were used to assess the cardiac systolic and diastolic function, respectively (n = 7–8). Heart samples were harvested and fixed with paraformaldehyde (n = 6). The HE staining (upper) and Masson’s trichrome staining (lower) were conducted to assess myocardial morphology and cardiac fibrosis (**d**). The relative area of myocardial fibrosis (**e**) was estimated (scale bar = 20 μm). qPCR analysis showed the increased expression of cardiac stress-related genes, including Nppa, Nppb, and Myh7 (**f**) in rats with DCM (n = 4), suggesting adverse ventricular remodeling. The MDA levels (**g**, nmol/mg) and DHE staining (**h**, **i)** of cardiac tissue were measured to reflect the increased oxidative stress in rats with DCM (scale bar = 40 μm, n = 7). *P < 0.05 versus normal group, #*P *< 0.05 versus DCM group, and *P *< 0.05 versus DM-BSMC group

The diabetes model was constructed as shown in the study scheme and timeline (Fig. 1A). Characterization of cultured BMSCs was identified by flow cytometry. BMSCs from health and diabetic rats expressed specific surface antigens CD29, CD90, and CD105, but did not express CD34, CD45, and CD11b (Additional file 1: Figure S1). We firstly generated DCM rat models induced by high-fat diet feeding and low-dose STZ injection, which was confirmed by decreased cardiac function, and increased expression of cardiac stress-related genes, including *Nppa, Nppb, and Myh7* (Fig. 1B–G). We compared the impact of BMSCs isolated from mice with diabetes and nondiabetes on the phenotypes of the DCM model. Echocardiography indicators suggested that BMSCs injection from both mice models improved cardiac remodeling and function, including LVEF, FS, LVIDD, LVIDS, and E′/A′, compared with DCM mice (Fig. 1B–D; Additional file 2: Figure S2). However, the effect of BMSCs derived from healthy mice on cardiac function was significantly better than that of diabetic rat-derived BMSCs. Pathological Masson staining of heart tissue showed that DCM was accompanied by obvious myocardial interstitial fibrosis. BMSCs derived from normal rats but not from diabetes donors significantly reduced the degree of fibrosis (Fig. 1E, F). RT-PCR analysis indicated that the expression of cardiac remodeling-related genes, including *Nppa, Nppb, and Myh7*, in the normal donor group was markedly decreased compared to that in the diabetic stem cell treatment group and the untreated group (Fig. 1G). Oxidative stress is one of the key factors in the progression of DCM. We also observed that BMSCs derived from normal rats but not derived from diabetes significantly reduced the level of MDA and ROS in cardiac tissue (Fig. 1H–J). Taken together, the above results indicate that BMSCs derived from normal rats significantly delay the pathophysiological progress of DCM, and the therapeutic effect was superior to the stem cells derived from diabetic donors.

### In vitro evaluation of the phenotypic differences between normal-derived BMSCs and diabetes-derived BMSCs

**Fig. 2:**
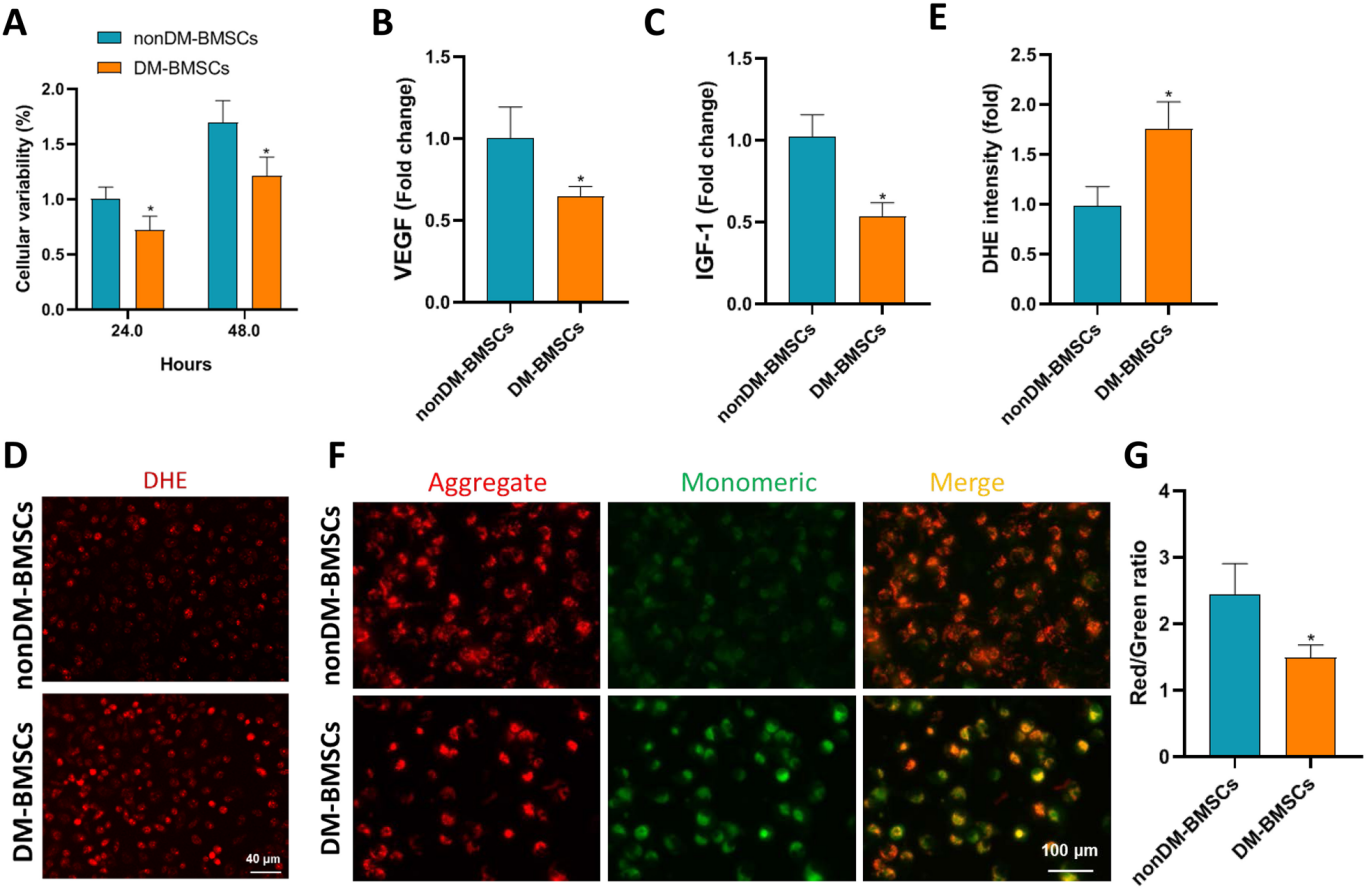
The comparison of cellular properties between normal and diabetic BMSCs. The CCK-8 assay (**a**) showed that the cellular viability of DM-derived BMSCs was significantly worse than that of non-DM rats (n = 6). The levels of VEGF (**b**) and IGF-1 (**c**) in the medium were measured to reflect the paracrine function of BMSCs (n = 5). According to the DHE staining (**d**, **e**) and JC-1 staining (**f**, **g**), diabetic BMSCs had higher ROS level and mitochondrial dysfunction than nondiabetic BMSCs (n = 5–6). *P < 0.05 versus nondiabetic BMSCs group.

Because normal- and DM-derived stem cells have significant differences in the therapeutic effects of DCM in vivo, we further explored in vitro whether the functions of the two stem cells are different. As expected, the cellular viability of DM-derived BMSCs was significantly worse than that of non-DM rats (Fig. 2A). Further analysis showed that the paracrine function of diabetic BMSCs was significantly weakened, including the lower levels of VEGF and IGF-1 (Fig. 2B, C), which may be one of the reasons for the weakened effect of diabetic BMSCs on DCM. Previous studies have shown that redox disorder is a key hallmark of stem cell aging. We further evaluated the oxidative stress-related markers of diabetic BMSCs. Compared to BMSCs from nondiabetic rats, stem cells derived from diabetic mice had significantly higher ROS levels (Fig. 2D, E). Mitochondrial dysfunction is the main source of oxidative stress. We found significantly reduced mitochondrial membrane potential in the DM-BMSCs group compared to healthy control (Fig. 2F, G). Thus, diabetes accelerated the aging process of BMSCs and thus compromised the therapeutical effect on DCM.

### Diabetes inhibiting the SAHH expression of stem cells may be a critical trigger factor for BMSCs aging

**Fig. 3:**
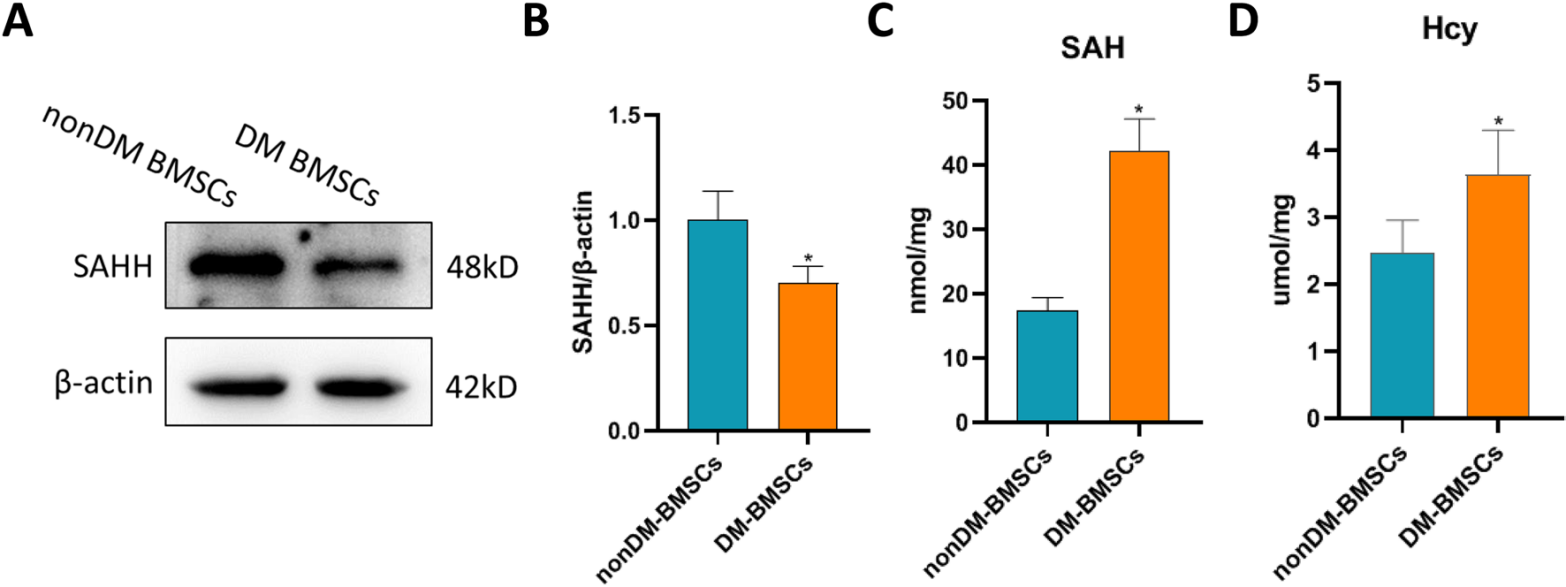
SAH metabolism in normal and diabetic BMSCs. Diabetes BMSCs had a decreased expression of SAHH protein (**a**, **b**) compared with the nondiabetic BMSCs (n = 4). The increased metabolites SAH (**c**, nmol/mg) and Hcy (**d**, μmol/mg), especially SAH, in diabetes BMSCs also suggested an insufficient SAHH activity (n = 5). *P < 0.05 versus nondiabetic BMSCs group.

Previous studies have suggested that SAHH plays a key role in maintaining mitochondrial redox homeostasis. We found that diabetes status induced a decreased expression of SAHH protein in the BMSCs (Fig. 3A). SAHH-related metabolites SAH and Hcy, especially SAH, increased significantly in diabetic BMSCs compared with the nondiabetic cells (Fig. 3B, C). These results indicate that the up-regulated oxidative stress may be attributed to the decrease of SAHH expression in the BMSCs of the DM group.

### The effect of SAHH inhibition on the BMSCs derived from normal rats in vitro

**Fig. 4:**
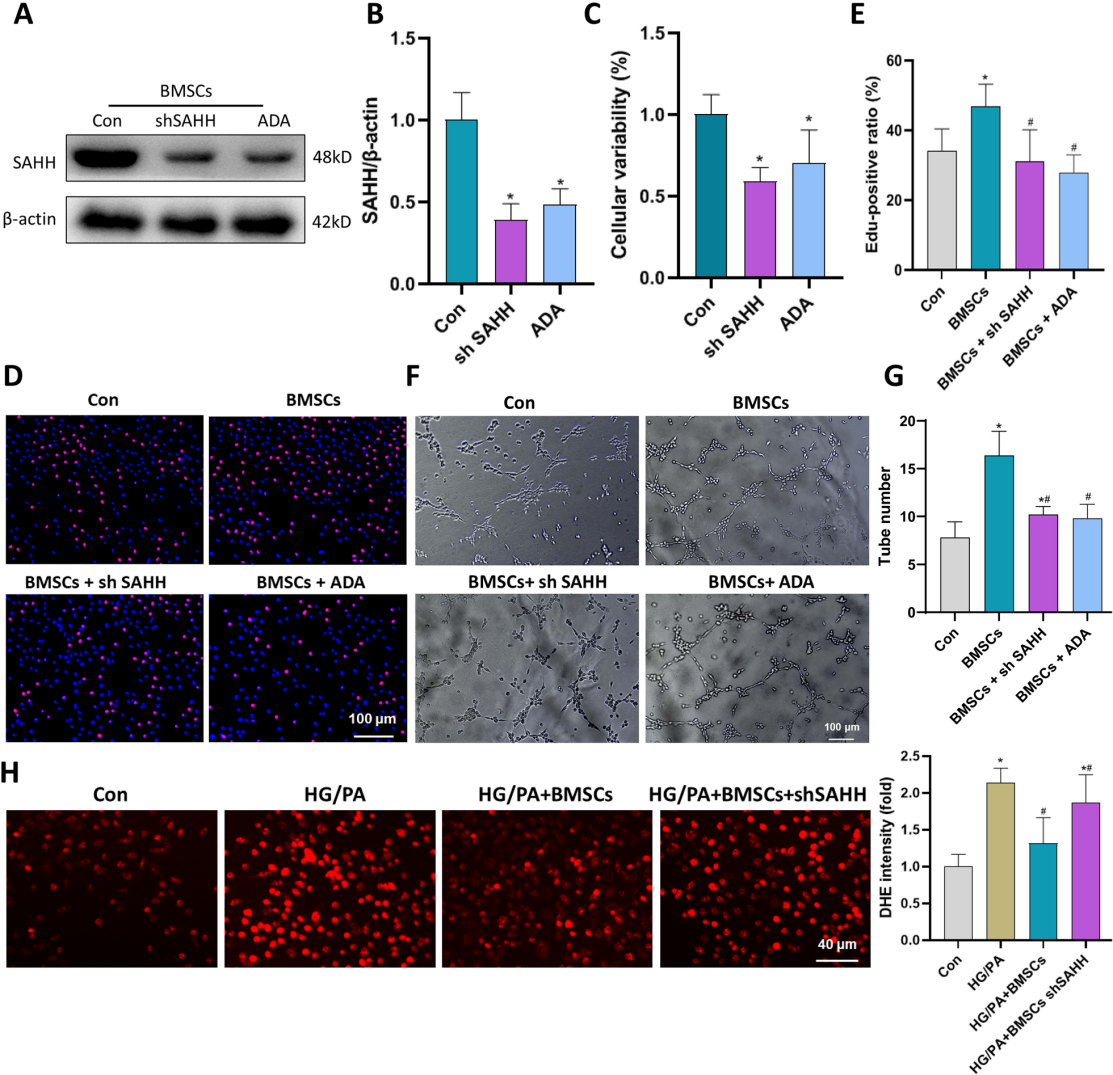
The effect of SAHH inhibition in the normal BMSCs on endothelial cells. Western blots showed that plasmid containing SAHH shRNA and ADA significantly reduced the expression of SAHH in normal BMSCs (**a**, **b**, n = 5). According to the CCK-8 assay (**c**), SAHH inhibition reduced the cellular variability of BMSCs (n = 5). *P < 0.05 versus normal group. After SAHH inhibition, nondiabetic BMSCs could not promote endothelial proliferation (**d**, **e**) and tube formation (**f**, **g**, n = 5). *P < 0.05 versus normal group, #*P *< 0.05 versus BMSCs group. ROS generation (**h**, **i**) in endothelial cells under high glucose and fatty acids was measured using DHE staining after treatment with or without medium derived from normal BMSCs (n = 5). *P < 0.05 versus normal group, #*P *< 0.05 versus HG/PA group, and *P *< 0.05 versus HG/PA +BMSCs group.

Next, we observed whether the inhibition of SAHH reduced the cellular variability and therapeutic effect of normal rat-derived BMSCs through pharmacological or genic intervention. Consistent with prior studies [22], both SAHH shRNA and ADA significantly reduced the expression of SAHH in normal BMSCs (Fig. 4A, B). The inhibition of SAHH significantly reduced the cellular variability of BMSCs (Fig. 4C). Next, we observed the effect of inhibiting the SAHH activity of BMSCs on endothelial cells in vitro. SAHH inhibition abolished the beneficial effect of normal rat-derived BMSCs to promote endothelial proliferation and tube formation (Fig. 4D–G). Furthermore, we simulated DCM status using endothelial cells cultured under high glucose and fatty acids conditions. The culture medium derived from normal BMSCs reduces the endothelial ROS level in endothelial cells, while the medium from BMSCs treated by SAHH shRNA had an insignificantly protective role in endotheliocytes (Fig. 4H, I). Thus, SAHH activity was critical for the therapeutical effect of health BMSCs.

### The effect of SAHH overexpression on the BMSCs derived from DM rats in vitro

**Fig. 5:**
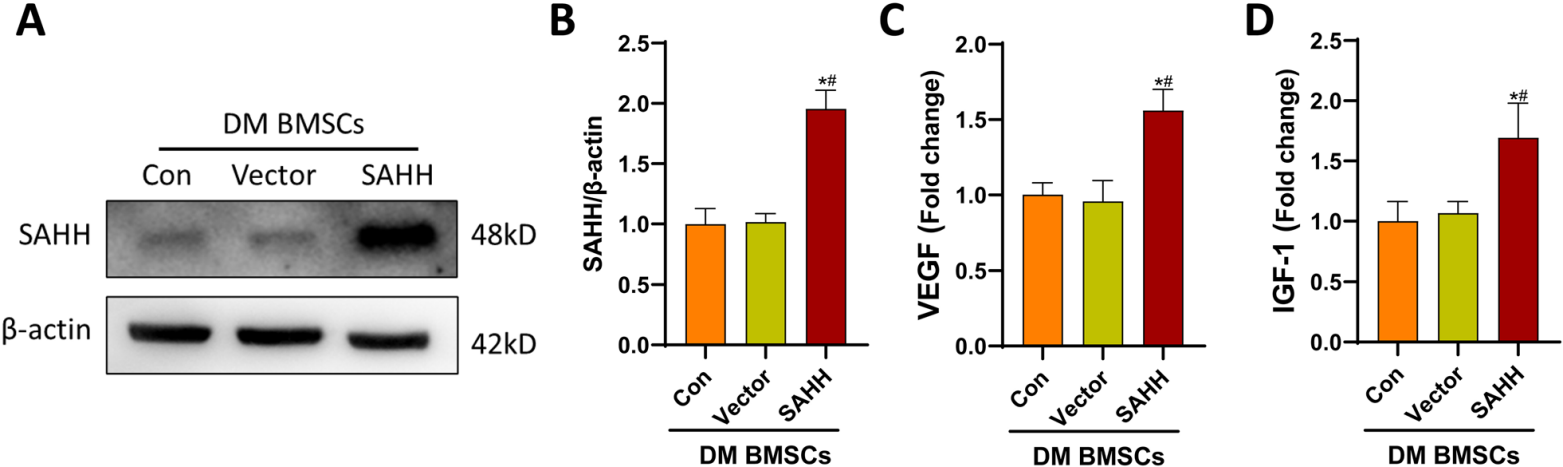
SAHH overexpression improved cellular activity of diabetic BMSCs . The plasmid containing the SAHH gene was transfected into diabetic BMSCs. 48 h later, the protein expression of SAHH in diabetic BMSCs was assessed by western blots (**a**, **b**, n = 4). The levels of VEGF (**c**) and IGF-1 (**d**) in the medium were measured to reflect the paracrine function of BMSCs (n = 5). *P < 0.05 versus control group, #*P *< 0.05 versus scramble (con) transfection group.

The source of BMSCs for clinical treatment of DCM remains most likely to be derived from diabetic patients themselves. Therefore, the modification of stem cells from diabetes to improve cell activity and function is worth studying. Based on the above results, we hypothesize that SAHH overexpression is beneficial to restore the activity of diabetic BMSCs. SAHH overexpression increased the protein content of SAHH and decreased cellular SAH levels in diabetes-derived BMSCs (Fig. 5A, B). As expected, SAHH up-regulation recovered the decreased paracrine function of diabetic BMSCs, suggesting that SAHH was a critical molecular to delay stem cell senescence under diabetic status (Fig. 5C, D).

### Transplantation of SAHH-overexpressing BMSCs improves the cardiac remodeling and angiogenesis for DCM rats in vivo

**Fig. 6:**
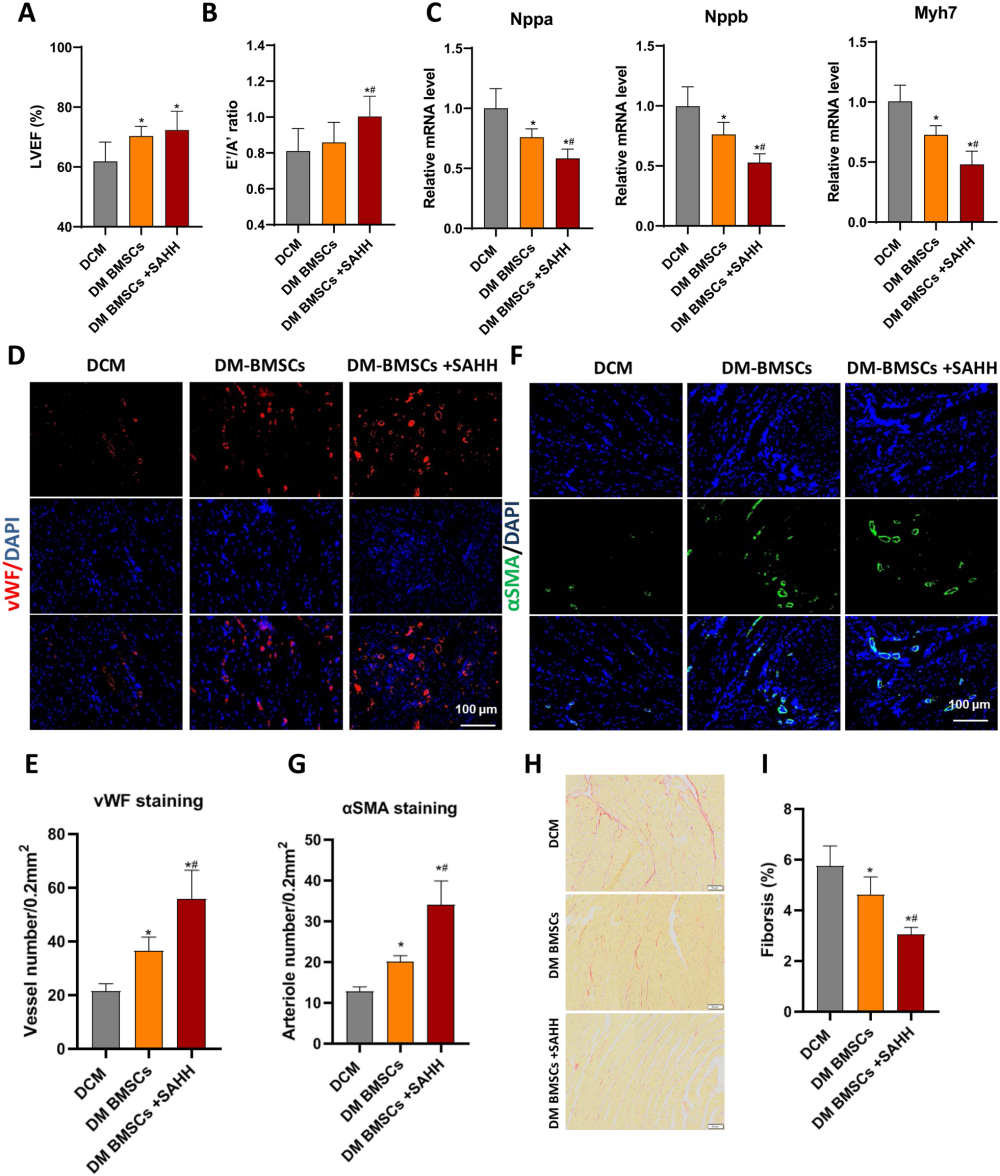
SAHH overexpression improves cardioprotection of diabetic BMSCs. We further evaluated whether the overexpression of SAHH in BMSCs derived from diabetic rats can improve the therapeutic value of stem cells to DCM. Diabetic BMSCs with SAHH overexpression were infused 4 times. The LVEF (**a**) and E’/A’ ratio (**b**) were used to assess the cardiac function using echocardiography (n = 8). qPCR analysis showed the increased expression of cardiac stress-related genes, including *Nppa, Nppb, and Myh7* (**c**) in rats with DCM (n = 4). Heart samples were harvested and fixed with paraformaldehyde. The immunofluorescence staining of vWF (**d**, **e**) and αSMA (**f**, **g**) was conducted to assess the pro-angiogenesis induced by BMSCs transplantation (n = 5). Sirius Red staining (**h**, **i**, n = 6) was conducted to assess to reflect myocardial fibrosis (scale bar = 100 μm). *P < 0.05 versus DCM group, #*P *< 0.05 versus DCM receiving diabetic BMSCs group.

We further evaluated whether the overexpression of SAHH in BMSCs derived from diabetic rats can improve the therapeutic value of stem cells to DCM. According to echocardiography examination, compared to diabetic BMSCs transplantation, SAHH-overexpressed diabetic BMSCs significantly promoted the improvement of heart diastolic function, although the difference in ejection fraction was moderate (Fig. 6A, B). Consistently, we also noted the decreased expression of *Nppa, Nppb, and Myh7* after transplantation of SAHH-overexpressing BMSCs (Fig. 6C). The vWF- and α-smooth muscle actin-positive (αSMA)-positive arteriole densities in the heart tissue were significantly higher in the SAHH-overexpressing BMSCs injection group compared with the diabetic BMSCs group (Fig. 6D–G). We measured the levels of EGFR and IGF in heart tissue. Cardiac VEGF not IGF-1 moderately increased after treatment with diabetic BMSCs, while diabetic rats receiving SAHH-overexpressed diabetic BMSCs had higher levels of VEGF and IGF than diabetic rats with diabetic BMSCs transplantation (Additional file 3: Figure S3). The collagen deposition in the hearts of the SAHH-overexpressing BMSCs group was moderately decreased compared to the diabetic BMSC-treated group (Fig. 6H, I). The above data demonstrated that SAHH overexpression in diabetes-derived BMSCs ameliorated cardiac dysfunction and fibrosis of DCM.

### SAHH overexpression activates Nrf2-mediated antioxidant signal and improves the biological activity of BMSCs derived from diabetic rats

**Fig. 7:**
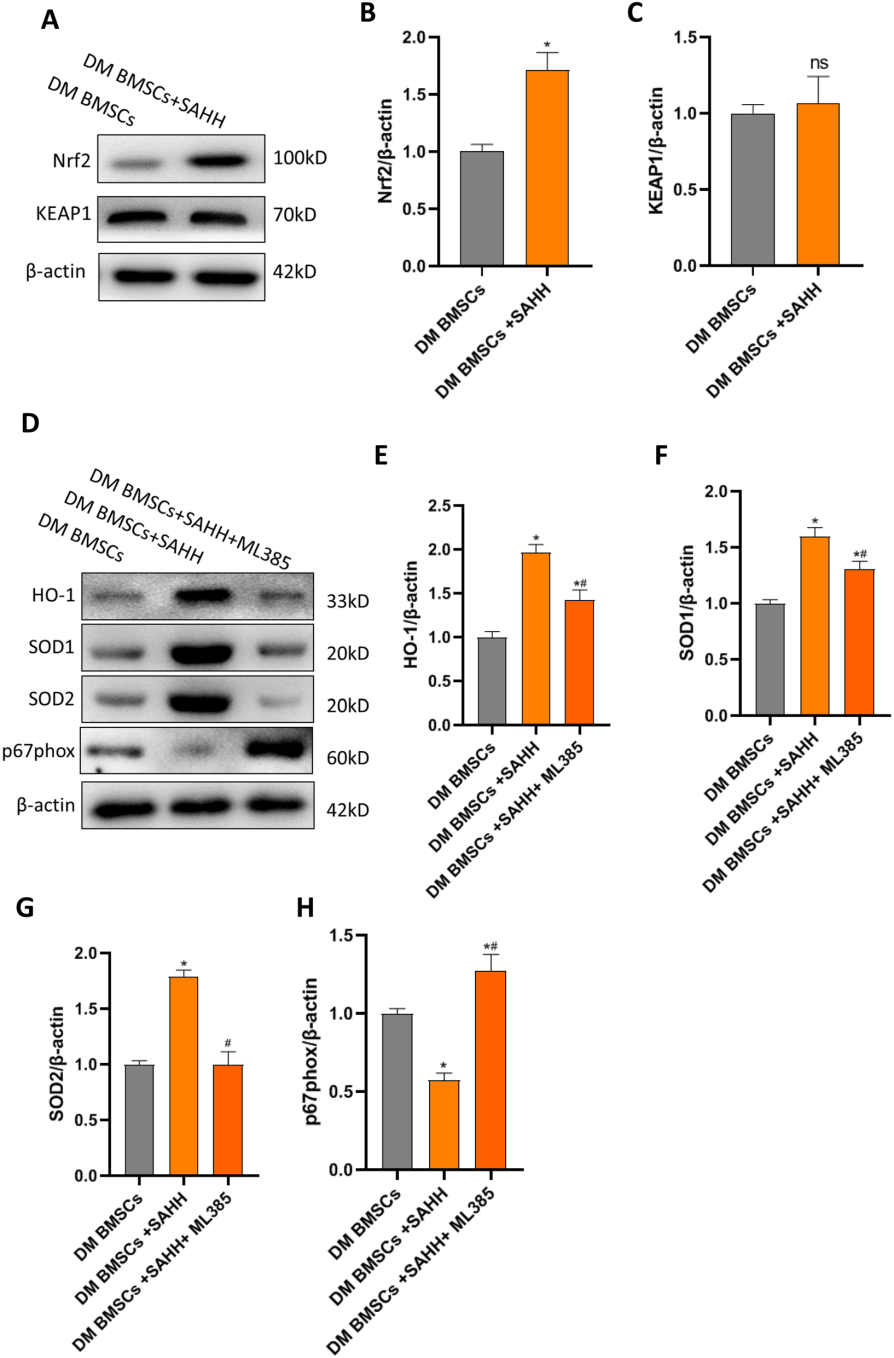
SAHH improves the biological activity of BMSCs partly by activating the Nrf2-mediated antioxidant signal . To further explore the underlying mechanism, we further explored whether SAHH activates the Nrf2-mediated antioxidant pathway to improve the activity and function of diabetic BMSCs. in vitro. The protein expression of Nrf2 and Keap1 was assessed in diabetic BMSCs after SAHH overexpression (**a**–**c**). After further treatment with ML385 (a Nrf2 inhibitor), the downstream molecules including anti-oxidative HO-1 (**d**, **e**), SOD1 (**f**), SOD2 (**g**), and pro-oxidative p67phox (**h**) were measured by western blots (n = 4). *P < 0.05 versus DM-BMSCs group, #*P *< 0.05 versus DM-BMSCs +SAHH group.

The activation of the Nrf2 antioxidant pathway is considered to be a critical protective signal for cellular survival under oxidative stress. We further explored whether SAHH activates the Nrf2-mediated antioxidant pathway to improve diabetic BMSC activity and function. SAHH overexpression improved the expression of Nrf2, while the expression of Keap1, an inhibitor of Nrf2 binding, did not change significantly (Fig. 7A–C). Therefore, SAHH may improve the phenotype of BMSCs by promoting the expression of Nrf2 not inhibiting Keap1. As expected, overexpression of SAHH up-regulated the protein expression of HO-1, SOD1, and SOD2 and reduced cellular p67phox and ROS levels (Fig. 7D–H). However, the Nrf2 inhibitor ML385 significantly abolished the up-regulated antioxidant signal and reduced oxidative stress induced by SAHH overexpression (Fig. 7D–H). The above data show that SAHH was very important to improve the cell viability and biological functions of diabetes-derived BMSCs, partly by regulating the Nrf2-mediated antioxidant signal pathway.

## Discussion

In this study, we investigated the distinctive effect of BMSCs harvested from health and diabetic donor rats on the diabetic heart. We found diabetic BMSCs had decreased therapeutic effects in the HFD- and STZ-induced DCM rats compared with nondiabetic BMSCs. Mechanistically, BMSCs derived from diabetic rats were more vulnerable to aging and had a lower expression of SAHH than BMSCs derived from normal rats. Further, we demonstrated that SAHH inhibition significantly weakened the favorable effects of normal BMSCs on diabetic cardiomyopathy, while the transplantation of diabetic BMSCs with SAHH overexpression promoted the recovery of heart function in diabetic cardiomyopathy partly by the activation of Nrf2-mediated antioxidant signal. To the best of our knowledge, this is the first study to provide a novel mechanistic insight into SAH-related improvement of stem cell activity and function in diabetes sitting.

Diabetes is a strong risk factor for oxidative stress, leading to abnormal stem cell physiological state and microenvironment. Accumulated evidence suggested that diabetes is involved in mitochondrial dysfunction, impaired lipid and glucose oxidation, and excessive production of reactive oxygen species (ROS) [16, 27, 29]. The proliferation and differentiation of stem cells derived from skeletal muscle, nervous system, and adipose tissue are significantly attenuated in the diabetes status [9, 16, 30, 31]. Consistently, our study also demonstrated stem cells harvested from diabetes donors had impaired proliferation capability and faded therapeutic effect on diabetic cardiomyopathy [16]. Furthermore, our data explained that the clinical therapeutic effect of autologous stem cell transplantation in diabetic patients is limited, and thus, some measures need to be taken to improve the biological activity of diabetic BMSCs. Research on the treatment of cardiovascular disease by mesenchymal stem cell transplantation has been carried out in adults, but the therapeutic effect is not stable and consistent in clinical trials [32]. Part of the reason may be the reduced stem cell activity under pathological conditions, so it is difficult to repeat the conclusions in preclinical studies.

Consistent with our reports, several studies demonstrated that BMSCs from aging donors also had compromised repair ability [23, 33]. Huang et al. found that extracellular vesicles secreted by adipose-derived mesenchymal stem cells, derived from young mice but not aged mice, alleviated LPS-induced acute lung injury by mediating macrophage phenotypes and macrophage recruitment [23]. Tashiro et al. also demonstrated that young MSCs but not aging MSCs inhibited bleomycin-induced pulmonary fibrosis in the mice model [33]. Diabetes induced oxidative stress and thus was considered pathogenesis for biological aging [34]. Our findings suggested that limited benefits should be warranted regarding the clinical use of diabetic stem cell transplantation, and it is necessary to explore strategies to improve the physical viability and aging phenotype of diabetes-derived MSCs.

S-adenosylhomocysteine, the precursor of homocysteine, is hydrolyzed to homocysteine and adenosine under the catalysis of SAH hydrolase (SAHH) [35]. SAHH inhibition by pharmacological or genetic methods significantly advanced the development of atherosclerosis and impaired endothelium-dependent vascular relaxation via epigenetic up-regulation of the p66shc-mediated oxidative stress pathway [22]. Under diabetic sittings, a recent study found that inhibition of SAHH activity aggravated the process of diabetic nephropathy partly by inducing NLRP3 inflammasome activation and oxidative stress [35]. Xiao et al. recently reported that SAHH inhibition increased methylation in the p66shc gene promoter and ROS generation through inhibiting DNA methyltransferase 1 [22]. In our experiments, SAHH is one of the critical contributors to the phenotypic difference between diabetic and nondiabetic stem cells. Other and our results supported that SAHH is essential for redox homeostasis in nondiabetes and diabetes sittings. Overexpression of SAHH significantly improves the biological activity and function of diabetic BMSCs. Diabetes results in the insufficient expression and activity of SAHH, which further leads to an inhibition of Nrf2-mediated antioxidant capacity. And thus, BMSCs from diabetes donors were more susceptible to oxidative stress impairment and senescence, which reduces the ability to repair diabetic cardiomyopathy [16]. Our findings provide a novel insight into the role and mechanism of SAHH inhibition in diabetic stem cells. Activating SAHH activity in BMSCs to assist stem cell heart transplantation may be a potential strategy to improve the efficacy of stem cell therapy.

Mesenchymal stem cell transplantation therapy has been considered a promising alternative to cardiovascular disease [17]. Accumulative studies have demonstrated the efficiency of stem cell therapy in cardiometabolic disorders via regulating immunomodulatory, anti-inflammation, and multi-lineage potential [20]. The cardioprotective mechanism of stem cells is mainly attributed to the repair of tissue by paracrine cytokines and vesicles and even directly differentiates into functional cells such as endothelial and cardiac muscle. Our study aimed to elucidate the inconsistent conclusions of stem cell therapy in clinical studies and explored the underlying mechanisms. It is worth noting that, among previous studies of stem cell therapy in DCM rats, the source of stem cells was derived from young healthy donors, but the benefits and risks of allogeneic stem cell transplantation for nonhematopoietic diseases were still controversial. Autologous stem cells are still the most likely to be clinically translated. To the best of our knowledge, this study firstly compared the benefits of diabetic and nondiabetic-derived stem cell long-term infusion and found significantly different effects on DCM phenotypes. The cardioprotective effects and mechanisms of mesenchymal stem cells may be systemic. Other and our previous studies have reported that stem cells paracrine multiple factors involved in angiogenesis, including VEGF and IGF-1, microRNAs, as well as ornithine metabolites that were recently identified by untargeted metabolomics [5, 20, 36]. Additionally, our recent studies found that human decidual stem cells had a favorable capacity to differentiate into vascular endothelial cells in the heart post-infarction [36]. In this study, although cytokines VEGF and IGF-1 were not stem cell-specific, we found that heart tissues had higher contents of VEGF and IGF-1 in DCM rats with SAHH-overexpressed diabetic BMSCs transplantation than diabetic BMSCs. Moreover, we observed diabetic BMSCs secreted lower levels of cytokines VEGF and IGF-1 than nondiabetic BMSCs, suggesting a decreased function of paracrine in diabetic BMSCs. Further studies should be investigated to uncover the underlying mechanisms. Nonetheless, our study highlights that SAHH inhibition is one of the key causes of redox disturbances in diabetic BMSCs and results in compromised therapeutic effects. Targeting the SAHH activity of diabetic stem cells may be a potential strategy to improve the activity of diabetic stem cells.

## Conclusions

Diabetes leads to compromised cellular activity and cardiac repair capacity of BMSCs. The intravenous infusion BMSCs derived from health rats but not diabetic rats ameliorated the progression of diabetic cardiomyopathy, which was partly attributed to the inhibited SAHH/Nrf2 signals and redox disorder in diabetic BMSCs. Our study suggests that SAHH activation may improve the cardioprotective effect of autologous transplantation of diabetes-derived BMSCs on patients with DCM.

## Supplementary Information

**Additional file 1. Figure S1:** Identification of BMSCs harvested from diabetic rats. Phenotypical identifications of BMSCs were assessed by flow cytometry

**Additional file 2. Figure S2:** The effect on cardiac function of diabetic and nondiabetic BMSCs. Cardiac function was measured using echocardiography, including (a) Measurement of LVFS (%); (b) Measurement of LVIDD (cm); (c) Measurement of LVIDS (cm). * p<0.05 vs. Con group, #P < 0.05 vs. DCM group, and P < 0.05 vs. DCM treated with nondiabetic BMSCs (n=7-8).

**Additional file 3. Figure S3:** Cardiac contents of VEGF and IGF-1 in DCM rats after treatment with diabetic BMSCs. Cardiac VEGF and IGF-1 in rats with DCM were measured using ELISA kits 4 weeks after BMSC infusion (n=8). * p<0.05 vs. DCM group, #P < 0.05 vs. DCM rats treated with DM-BMSCs.

**Additional file 4. Table S1:** Primer sequences for qRT-PCR.

## Data Availability

Data and study materials are available.

## Abbreviations

DCM: Diabetic cardiomyopathy
BMSCs: Bone marrow mesenchymal stem cells
STZ: Streptozotocin
SAH: S-adenosylhomocysteine
SAHH: S-adenosylhomocysteine hydrolase
T2DM: Type 2 diabetes mellitus
BMSCs: Mesenchymal stem cells
HFD: High-fat diets
STZ: Streptozotocin
ADA: Adenosine deaminase
HUVECs: Human umbilical vein endothelial cells
PA: Palmitic acid
LVIDd: Left ventricular end-diastolic diameter
LVISd: Left ventricular end-systolic diameter
LVEF: Left ventricular ejection fraction
LVFS: Left ventricular shortening fraction
MDA: Malondialdehyde
ROS: Reactive oxygen species
