# Supplementary figures and images for: Insufficient S-adenosylhomocysteine hydrolase compromises the beneficial effect of diabetic BMSCs on diabetic cardiomyopathy
Source: Stem Cell Res Ther. 2022 Aug 13;13:418. doi: 10.1186/s13287-022-03099-1 (PMC9375418; doi:10.1186/s13287-022-03099-1)

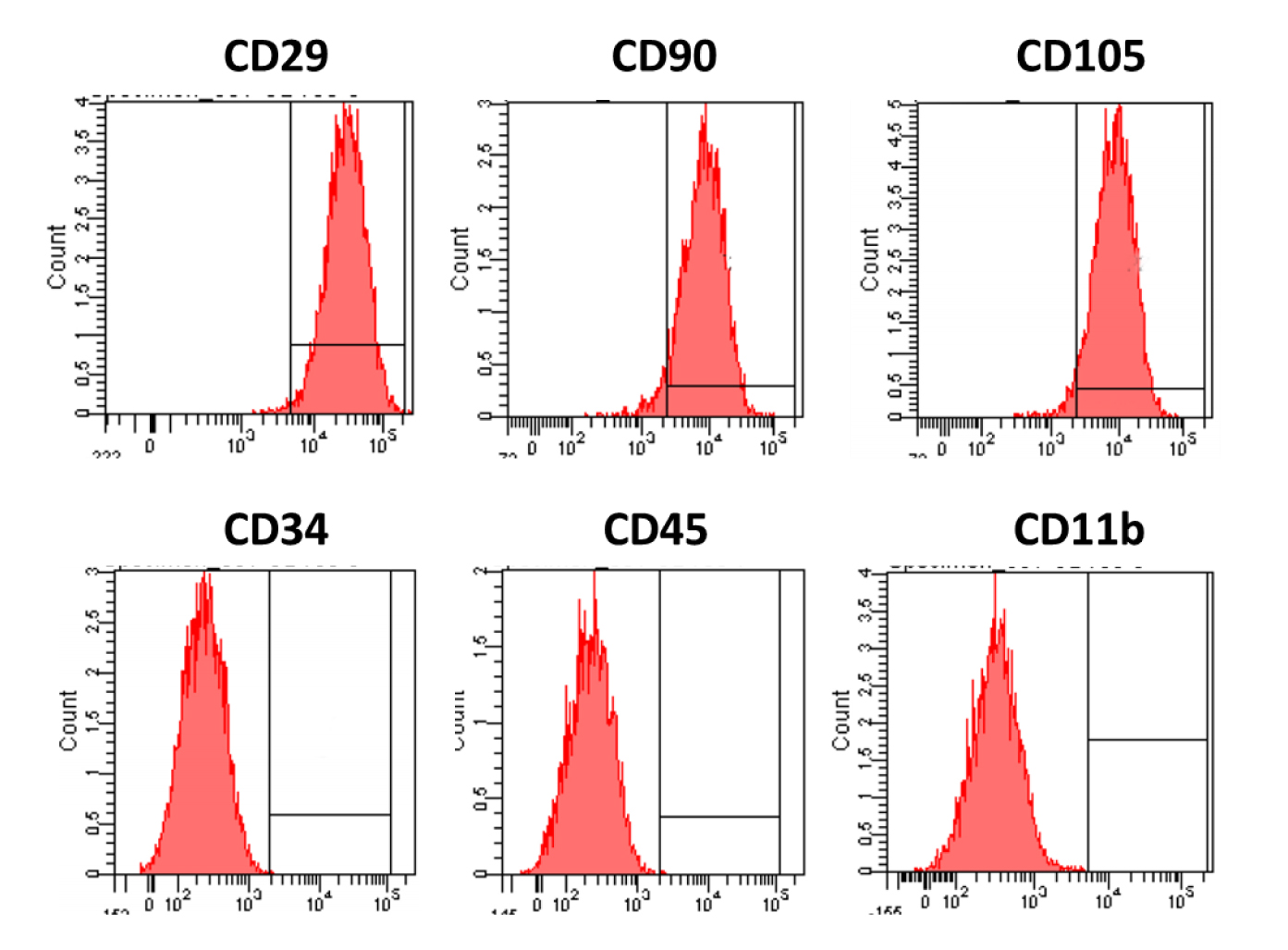

Supplement: Supplementary file 1 — Additional file 1. Figure S1: Identification of BMSCs harvested from diabetic rats. Phenotypical identifications of BMSCs were assessed by flow cytometry [file 13287_2022_3099_MOESM1_ESM.jpg]

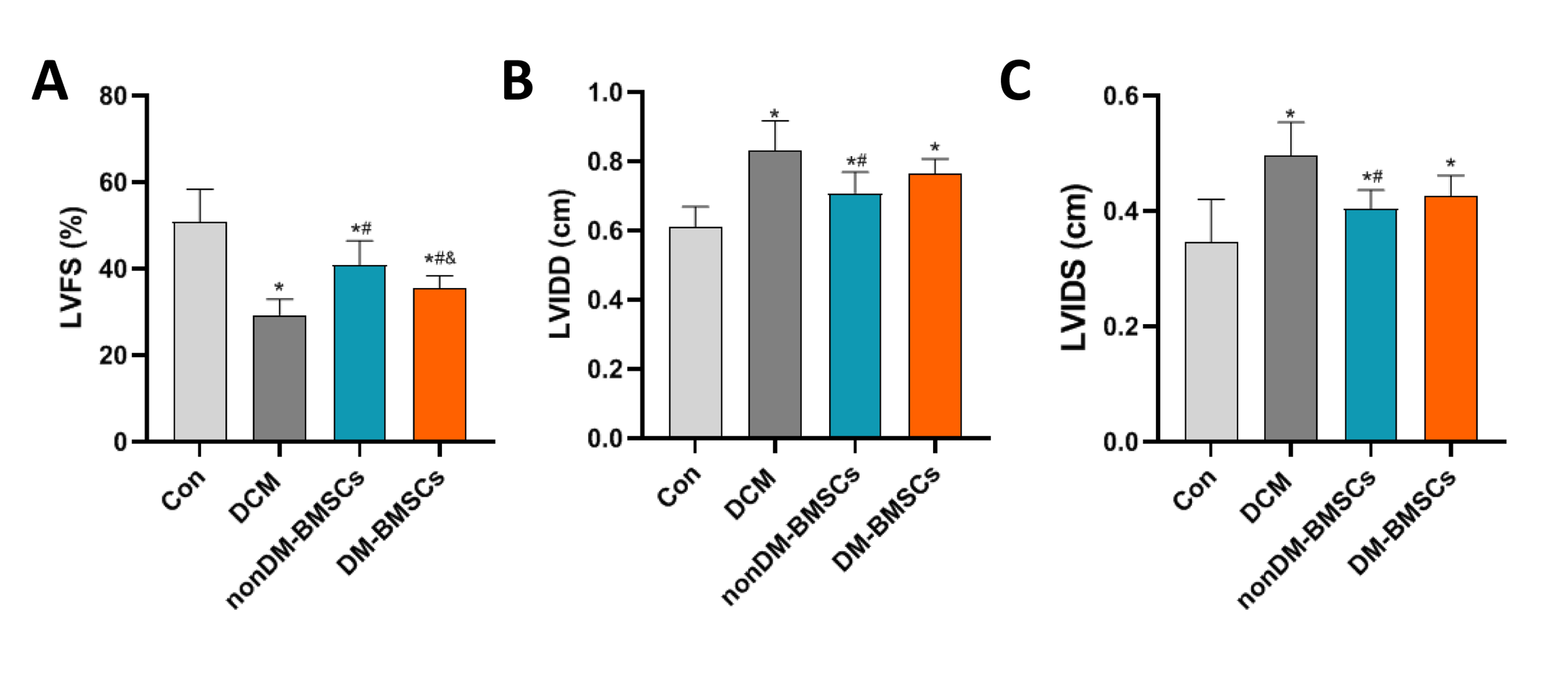

Supplement: Supplementary file 2 — Additional file 2. Figure S2: The effect on cardiac function of diabetic and nondiabetic BMSCs. Cardiac function was measured using echocardiography, including (a) Measurement of LVFS (%); (b) Measurement of LVIDD (cm); (c) Measurement of LVIDS (cm). * p<0.05 vs. Con group, #P < 0.05 vs. DCM group, and P < 0.05 vs. DCM treated with nondiabetic BMSCs (n=7-8). [file 13287_2022_3099_MOESM2_ESM.jpg]

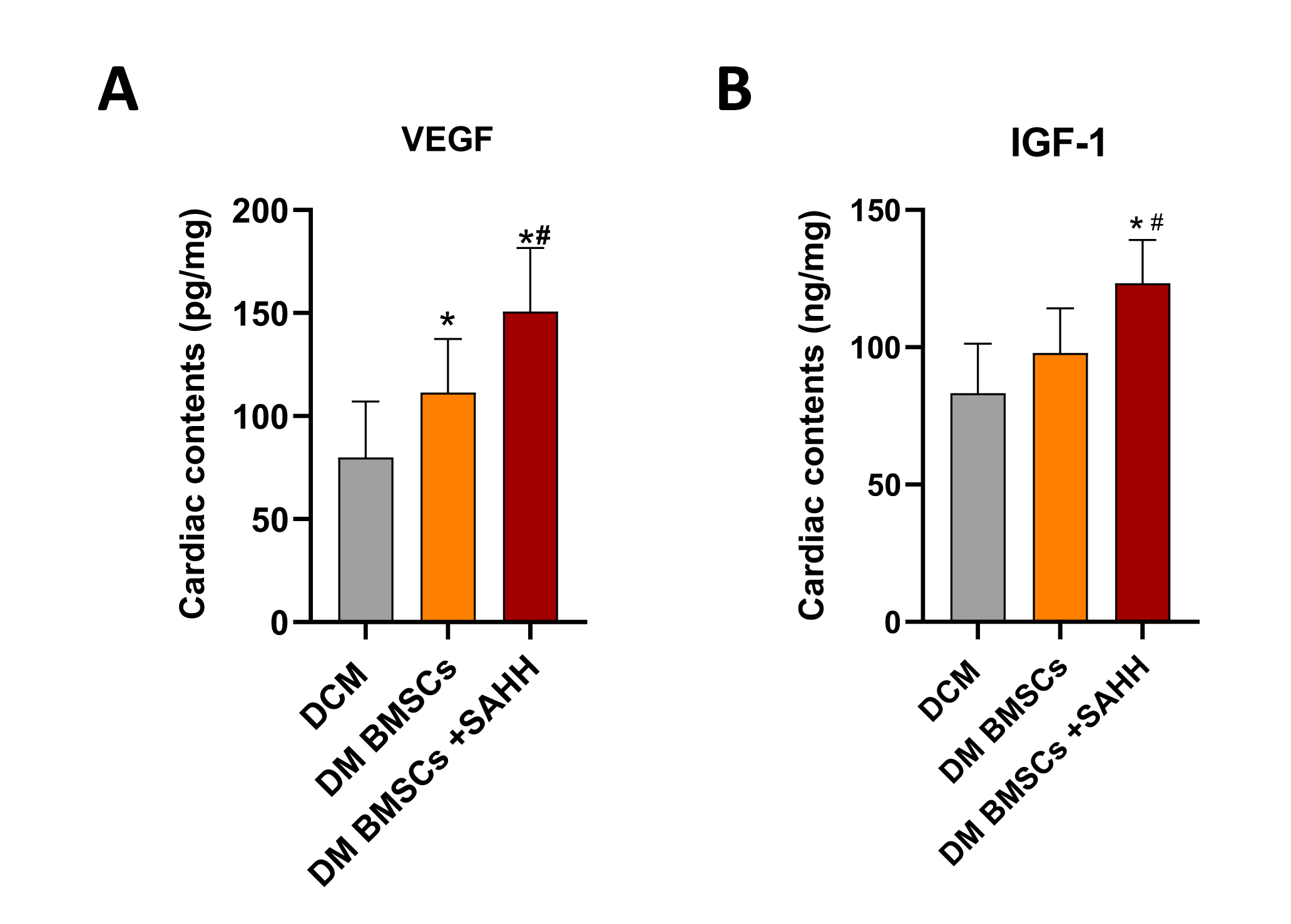

Supplement: Supplementary file 3 — Additional file 3. Figure S3: Cardiac contents of VEGF and IGF-1 in DCM rats after treatment with diabetic BMSCs. Cardiac VEGF and IGF-1 in rats with DCM were measured using ELISA kits 4 weeks after BMSC infusion (n=8). * p<0.05 vs. DCM group, #P < 0.05 vs. DCM rats treated with DM-BMSCs. [file 13287_2022_3099_MOESM3_ESM.jpg]
