# Supplementary material for: Insufficient S-adenosylhomocysteine hydrolase compromises the beneficial effect of diabetic BMSCs on diabetic cardiomyopathy
Source: Stem Cell Res Ther. 2022 Aug 13;13:418. doi: 10.1186/s13287-022-03099-1 (PMC9375418; doi:10.1186/s13287-022-03099-1)
Supplement: Supplementary file 4 — Additional file 4. Table S1: Primer sequences for qRT-PCR. [file 13287_2022_3099_MOESM4_ESM.docx]

## Table S1. Primer sequences for qRT-PCR

| **Gene** | **Sequence** |
| --- | --- |
| Nppa |  |
|  | Forward: CGGACAAAGGCTGAGAGAGA |
|  | Reverse: ACCGCACTGTATACGGGATT |
| Nppb |  |
|  | Forward: ACAATCCACGATGCAGAAGC |
|  | Reverse: TAGGGCCTTGGTCCTTTGAG |
| Myh7 |  |
|  | Forward: CCAACACCAACCTGTCCAAG |
|  | Reverse: CTCTCCTCTGCCTCATCCAG |
